# Complex Network Analysis of CA3 Transcriptome Reveals Pathogenic and Compensatory Pathways in Refractory Temporal Lobe Epilepsy

**DOI:** 10.1371/journal.pone.0079913

**Published:** 2013-11-21

**Authors:** Silvia Yumi Bando, Filipi Nascimento Silva, Luciano da Fontoura Costa, Alexandre V. Silva, Luciana R. Pimentel-Silva, Luiz HM. Castro, Hung-Tzu Wen, Edson Amaro, Carlos Alberto Moreira-Filho

**Affiliations:** 1 Department of Pediatrics, Faculdade de Medicina da Universidade de São Paulo (FMUSP), São Paulo, São Paulo, Brazil; 2 Instituto de Física de São Carlos, Universidade de São Paulo, São Carlos, São Paulo, Brazil; 3 Department of Biosciences, Universidade Federal de São Paulo, Santos, São Paulo, Brazil; 4 Clinical Neurology Division, Hospital das Clínicas da FMUSP, São Paulo, São Paulo, Brazil; 5 Epilepsy Surgery Group, Hospital das Clínicas da FMUSP, São Paulo, São Paulo, Brazil; 6 Department of Radiology, Faculdade de Medicina da Universidade de São Paulo (FMUSP), São Paulo, São Paulo, Brazil; Queen's University Belfast, United Kingdom

## Abstract

We previously described – studying transcriptional signatures of hippocampal CA3 explants – that febrile (FS) and afebrile (NFS) forms of refractory mesial temporal lobe epilepsy constitute two distinct genomic phenotypes. That network analysis was based on a limited number (hundreds) of differentially expressed genes (DE networks) among a large set of valid transcripts (close to two tens of thousands). Here we developed a methodology for complex network visualization (3D) and analysis that allows the categorization of network nodes according to distinct hierarchical levels of gene-gene connections (node degree) and of interconnection between node neighbors (concentric node degree). Hubs are highly connected nodes, VIPs have low node degree but connect only with hubs, and high-hubs have VIP status and high overall number of connections. Studying the whole set of CA3 valid transcripts we: i) obtained complete transcriptional networks (CO) for FS and NFS phenotypic groups; ii) examined how CO and DE networks are related; iii) characterized genomic and molecular mechanisms underlying FS and NFS phenotypes, identifying potential novel targets for therapeutic interventions. We found that: i) DE hubs and VIPs are evenly distributed inside the CO networks; ii) most DE hubs and VIPs are related to synaptic transmission and neuronal excitability whereas most CO hubs, VIPs and high hubs are related to neuronal differentiation, homeostasis and neuroprotection, indicating compensatory mechanisms. Complex network visualization and analysis is a useful tool for systems biology approaches to multifactorial diseases. Network centrality observed for hubs, VIPs and high hubs of CO networks, is consistent with the network disease model, where a group of nodes whose perturbation leads to a disease phenotype occupies a central position in the network. Conceivably, the chance for exerting therapeutic effects through the modulation of particular genes will be higher if these genes are highly interconnected in transcriptional networks.

## Introduction

Epilepsy prevalence ranges from 0.5–1.0% in developed countries to 1.4–5.7% in developing countries [1], [2]. Around 30% of epileptic patients are refractory to the available antiepileptic drugs [3] and account for 80% of epilepsy cost [4]. Mesial temporal lobe epilepsy (MTLE) is the most common cause of drug-resistant epilepsy [4], [5]. Prolonged febrile seizures in early childhood constitute an initial precipitating injury usually associated with MTLE [6]–[9]. About 40% of the patients with MTLE and a history of febrile seizures develop refractory epilepsy [10]. Surgical treatment for pharmacoresistant epilepsy has attained good results but only a minority of patients with medically refractory epilepsy are ever referred to epilepsy surgery – less than 1% in USA [11] – and often too late to prevent serious disabilities [11], [4]. This situation reinforces the demand for identifying genomic mechanisms underlying refractory epilepsy that can be targeted for novel preventive and drug-based therapeutic interventions [12]–[15].

The search for new drug-targets in epilepsy has been impacted by recent advances in functional genomics and systems biology [12]–[15]. Hippocampal explants obtained at epilepsy surgery constitute a valuable material for functional genomic studies because it is probable that epileptogenic genes and pathways remain active in chronic disease, since focal regions of the brain continue life-long chronically hyperexcitable [12]. Moreover, transcriptional analyses in humans and in animal models of chronic epilepsy portrait the disease as a disturbed network of gene-gene interactions [12], [13], [15], [16]. Therefore, the study of transcriptional networks in epileptic brain tissues could be very helpful for multi-target drug discovery [12]–[16].

We previously described – studying transcriptional signatures of hippocampal CA3 explants obtained at epilepsy surgery – that febrile (FS) and afebrile (NFS) forms of refractory mesial temporal lobe epilepsy (RMTLE) have different pathomechanisms and constitute two distinct genomic phenotypes as shown by CA3 transcriptional co-expression networks [16]. Ex vivo high resolution magnetic resonance imaging (MRI) texture analysis of dentate gyrus (DG) also differentiates FS and NFS cases [17] and histological examination revealed that FS cases display higher neuronal cell loss in the DG granule cell layer [16], [17]. Comparative analysis of CA3 transcriptional interaction networks revealed molecular mechanisms underlying RMTLE phenotypes and indicated potential therapeutic targets [16]. However, this network analysis was based on a limited number (hundreds) of differentially expressed genes (DE networks) among a large set of valid transcripts (close to two tens of thousands).

In transcriptional interaction networks, highly connected genes, or hubs, hold the whole network together and are either associated to specific cellular processes or link different biological processes, organizing the interactome [18]–[21]. Complex disease genes, such as those involved in RMTLE, usually display higher number of connections in transcriptional networks, being called broker genes because they connect many proteins that would not be otherwise connected [22]. Therefore, in order to acquire a better understanding on the molecular mechanisms underlying RMTLE genomic phenotypes it would important to develop methods to study the complete set of valid transcripts in a particular disease target tissue or cell population. The mathematical and computational tools for this task lay in the field of complex network analysis [21], [23].

Here we developed a methodology for complex network visualization and analysis that allows the categorization of network nodes according to distinct hierarchical levels of gene-gene connections, or node degree, and of interconnection between node neighbors, or concentric node degree (see Methods). We were thus able to study the whole set of CA3 valid transcripts in order to: i) obtain complete transcriptional networks, here called CO networks, for FS and NFS phenotypic groups; ii) examine how CO and DE networks are related; iii) identify genomic and molecular mechanisms underlying FS and NFS phenotypes, contributing for the discovery of potential targets for therapeutic interventions.

## Materials and Methods

### Patients

The RMTLE patients included in this study were selected through the CInAPCe-FAPESP Program (www.fapesp.br; www.cinapce.org.br). This research has been approved by Hospital das Clínicas da FMUSP and Hospital Albert Einstein, São Paulo, SP, Brazil institutional review boards under numbers 251/05 and CAEE 0122.0.028.174.05. A written informed consent was obtained from all patients. Refractory epilepsy cases were defined as those who have not gained seizure control after treatment with two or more antiepileptic drugs. All patients underwent preoperative clinical, electrophysiological, neuropsychological and neuroimaging evaluations. In this study we compared global gene expression profiles of CA3 explants obtained at surgery room from six FS and twelve NFS patients undergoing corticoamigdalohippocampectomy. None of these patients had mental retardation or first-degree family members with epilepsy or febrile seizure history. Detailed descriptions of the patient's demographic and clinical data and of the procedures for brain tissue collection and preservation for neuropathological (MRI and histopathology) and genomic studies (DNA microarrays) were published elsewhere [16].

### Brain tissue specimens for gene expression and neuropathological studies

Fresh ex-vivo explants from hippocampal CA3 of our patients were obtained in the operating room and immediately preserved with RNAlater (Qiagen cat. no. 76106, Valencia, CA). MRI and histological studies were performed in all removed hippocampi for neuropathology analysis and for confirming that the explants for genomic studies were obtained at the proper site [16], [17].

### RNA extraction

Brain tissue explants from CA3 (3–4 mm3) were homogenized with TissueRupter (Qiagen, cat. no. 9001272 Valencia, CA). Total RNA was extracted from the homogenates with RNeasy Lipid Tissue Kit (Qiagen cat. no. 74804, Valencia, CA) according to manufacturer's instructions. RNA quality was assessed on the Agilent BioAnalyzer 2100 (Agilent, Santa Clara, CA). All samples were stored at −80°C until used in hybridization experiments.

### Microarray hybridization and gene expression analysis

In order to determine gene expression profiles, 44 K DNA microarrrays (Whole Human Genome Microarray Kit, Agilent Technologies, cat no. G4112F, Santa Clara, CA) were used. The procedures for hybridization followed the protocols provided by the manufactureŕs instructions (One-Color Microarray-Based Gene Expression Analysis – Quick Amp Labeling). The images were captured by the reader Agilent Bundle according to the parameters recommended for bioarrays and extracted by Agilent Feature Extraction software version 9.5.3, considering spots with none or only one flag (i.e. low intensity, saturation, controls, etc.). The selected transcripts were used for analysis using the R software version 2.11.1 (R Development Core Team, 2010) and the Lowess test for arrays normalization. We identified 15,615 valid GO annotated genes for the CA3 samples (6 FS and 12 NFS patients). By means of the TMEV software version 4.6.1 [24] we obtained 307 differentially expressed GO annotated genes using the Significance Analysis of Microarrays (SAM) procedure, all up-regulated in the FS group (fold ≥3.0). All microarray raw data has been deposited in GEO public database (http://www.ncbi.nlm.nih.gov/geo) a MIAME compliant data base, under accession number GSE28674. Differential gene expression data were validated through quantitative real-time polymerase chain reaction [16].

### Transcriptional interaction network analysis

Transcriptional interaction networks for CA3 differentially expressed GO annotated genes (DE) and CA3 transcriptional interaction complex networks for all valid GO annotated genes (CO) were constructed for FS and NFS groups based on Pearsońs correlation. Pearson correlation identifies sets of genes which co-varies (positively or negatively), thus allowing us to construct networks by considering nodes as genes, with edges inferred if a pair presents high absolute value of correlation. Specifically, we define a correlation threshold that determines if edges are present or absent in the resulting network. This is done in a way that all nodes are connected to the major component and the network is stable in the sense that slight changes in the threshold value do not significantly affect its topological structure. It is an usual approach for constructing transcriptional interaction networks, also applied in other widely used tools (e.g. FunNet [25]). Data analysis and visualization (see below) were accomplished through the software developed by two of the authors, FNS and L da FC (http://cyvision.if.sc.usp.br/~bant/hierarchical/), which provides both dynamic and interactive data analysis of complex networks.

#### Concentric characterization of complex network analysis

While statistical measurements based directly on the degree present important information about the overall structure of complex networks, they fail to describe the local features of each node. The clustering coefficient [26] further extends the repertoire of complex networks measurement by considering the number of interconnections between the first neighborhoods of a node normalized by the maximum number of possible connections between them. However, the clustering coefficient may not be sufficient to distinguish between small triangles and real high clustered nodes. The node degree can be extended to consider distinct levels of hierarchy (concentric levels) from a node and obtain a measurement from each level [26]–[28].

**Figure 1:**
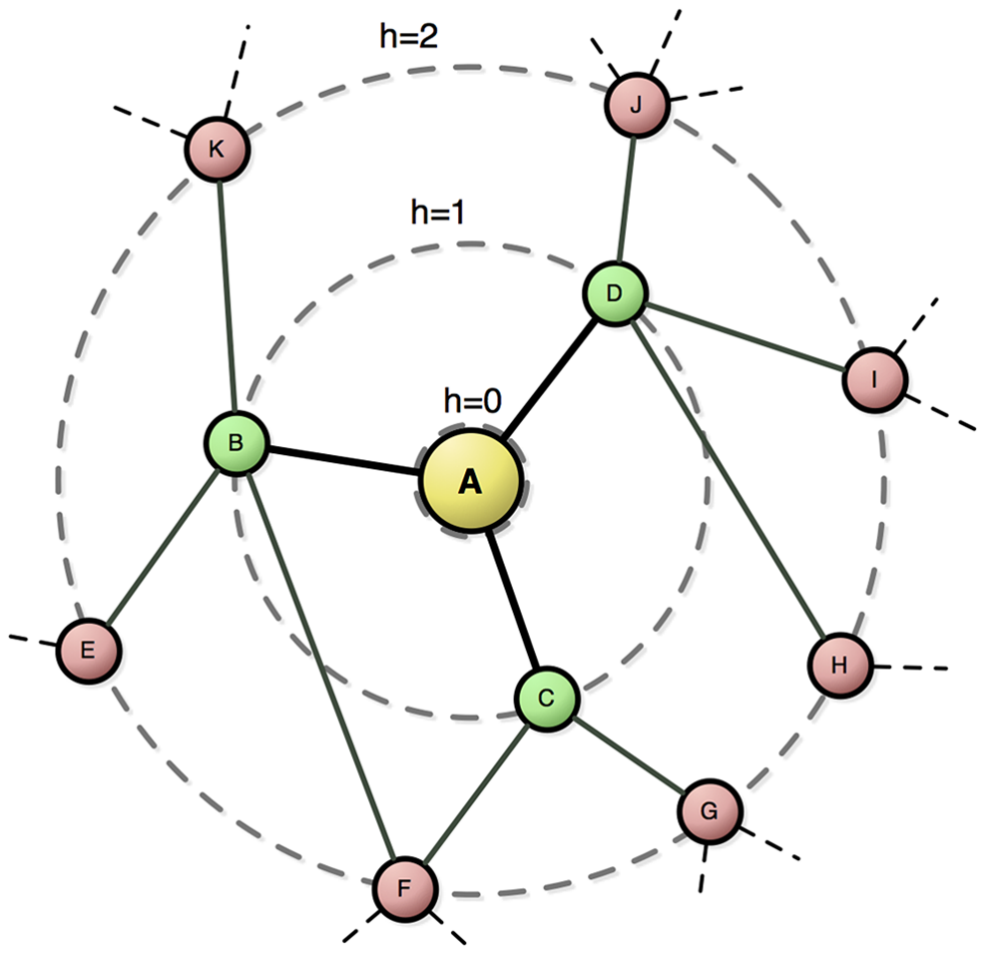
Concentric levels. Example of concentric levels of a network for node 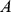 as reference (i.e. centered at node 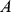). Each concentric level is represented by rings 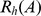, namely 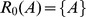, 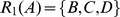 and 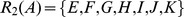, with concentric node degrees 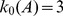 and 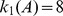.

Here we analyzed the networks by using the first neighborhood concentric degree and the usual node degree. This property is defined by means of concentric levels (or Rings),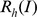, which represent the nodes at distance 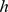 from a reference node 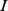. The concentric node degree,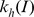, of level is defined as the number of connections between 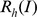 and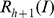. Figure 1 illustrates the concentric levels and measurements for a network example. All calculations were done by using the software available at http://cyvision.if.sc.usp.br/~bant/hierarchical/.

#### Hubs and VIPs

Traditionally, nodes with particularly high number of connections – the so-called called hubs – are known to play very important roles in most of real networks [21], [23]. However, in real networks, hubs may not strongly participate in some of the real networks dynamics. This is the case for controllability in gene regulatory networks where hubs tend to not become driven nodes (i.e. the network controllers) [29]. There is another node category that may play an important role on such networks: the VIPS, i.e. nodes presenting low node degree but mostly connected only with hubs [30]. In some networks VIPs may represent the highest control hierarchy in a system [20] and hubs may be shadowy influenced by VIPs [31]. Some nodes may present the VIP status for being connected with the majority of the hubs in a network, and also present high overall number of connections. Here we call these nodes high-hubs, which may play even more important roles than hubs and VIPs.

**Figure 2:**
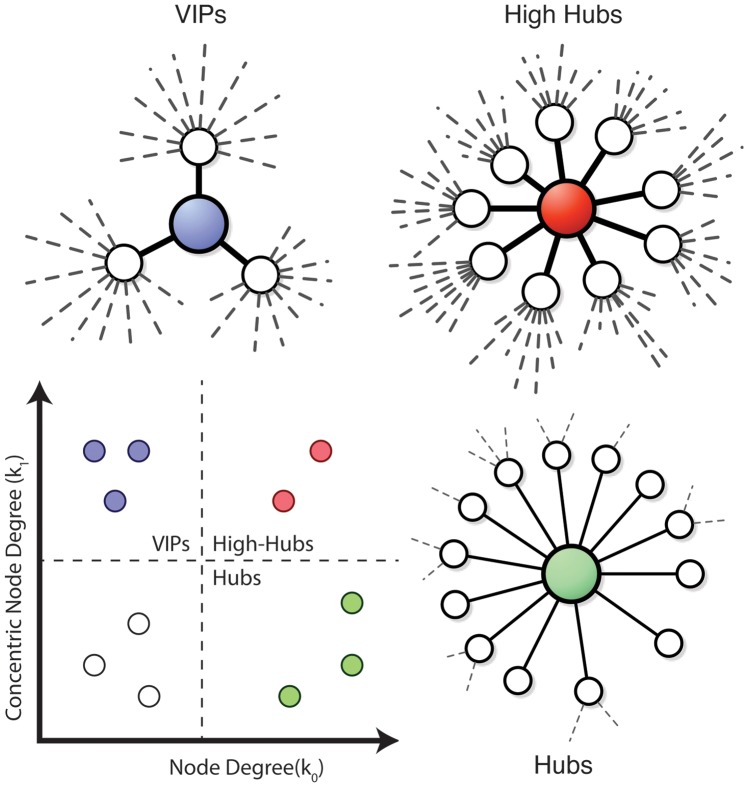
Node categories. Networks illustrating the three proposed categories of nodes with VIPs presenting low node degree but high concentric node degree at first level, hubs with lower concentric node degree and higher node degree, and high-hubs presenting high values of both properties.

One way to classify network nodes as VIPs, hubs or high-hubs is by obtaining the node degree, 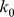, and the first level concentric node degree, 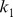, projecting all node values in a 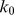 vs 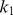 graphic. VIPs should present low 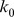 but high 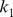, while hubs present high 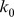 and low 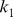, and high-hubs present high 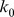 and 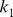 values. Figure 2 illustrates each of these node categories.

Because most real networks present scale-free distributions, there is no clear definition for setting a degree threshold for which we can classify nodes as being hubs or not [19], [21]. This same is true for objectively defining VIPs and high-hubs, since the distribution of 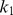 also suffers from the problem of not presenting a scale. Here we define hubs, VIPs and high-hubs by ranking them according to 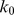 and 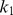, and then considering a set of those presenting the highest values of each property.

#### Betweenness centrality

Betweenness centrality [26], [32], [33] is a measurement of node importance which takes into account the entire set of shortest paths between nodes and passing through a particular node in a network. Betweenness is one of the most important topological properties of a network: nodes with the highest betweenness control most of the information flow in the network [20].

#### Network Visualization

Insights about the overall structure of complex networks can be obtained by projecting them into 2D or 3D spaces. This allows us not only to visualize but also to get a better understanding of which measurements and techniques may provide relevant information about these structures. In addition, inherent or calculated properties of nodes or edges can be interactively explored, providing more information than those obtained from statistical measurements such as moments and densities.

Complex networks can be visualized through projections of nodes represented as circles/balls and edges as lines/tubes connecting the nodes. Force-directed methods can be applied for visualization and are suitable for all kinds of networks, yielding visually pleasing drawings [33]–[35]. This method works by randomly placing the nodes over the metric space and incorporating physical forces between them, so that the structure relaxes to a minimal energy state.

We developed a software to obtain visualizations of large complex networks based on the Fruchterman-Reingold algorithm (FR) [35], which is a force-directed technique based on molecular dynamics employing both attractive and repulsive forces between nodes [36]. Each node repels each other by Coulomb forces resulting in a force 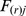 for each node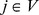, additionally, connected nodes, 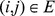, interact by a deviation of Hooke's Law, 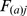, as described in Equations 1 and 2:
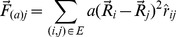
(1)

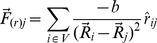
(2)where 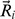 is the current position of node 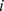 and 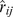 is the unit vector connecting the pair of nodes, with 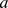 and 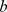 force constants.

By minimizing the energy of the system, one should obtain a set of positions for each vertex in a way that the preferred Euclidian distance between each connected pair is calculated by using Equation 3.
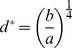
(3)


**Figure 3:**
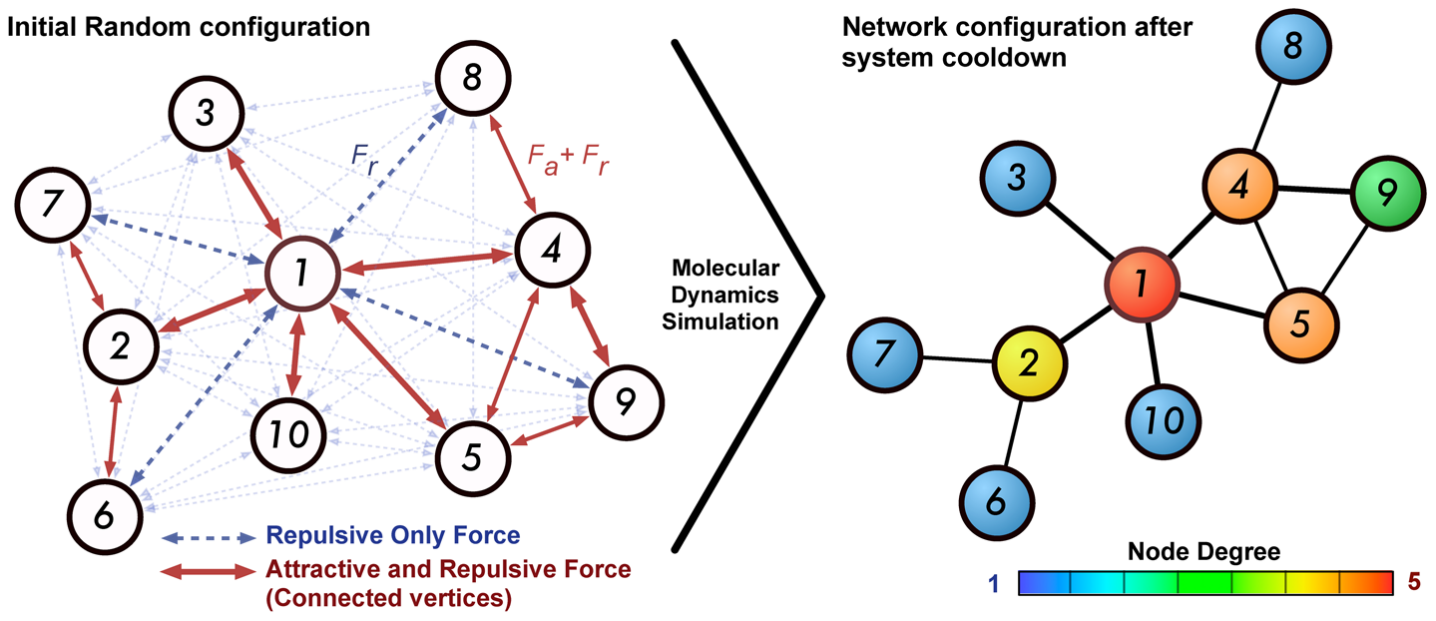
Network projection and visualization. On left side of the figure the network is randomly placed over the desired metric space (in this case, the 2D plane) and the forces described by equations (1) and (2) acting on each node are shown as arrows. Nodes without connection between them (displayed as fading blue arrows) interact only by repulsive forces while connected nodes (red arrows) present both attractive and repulsive interactions. Through the molecular dynamics simulation the system attains a lower energy state with connected nodes being close together if an edge is present and further away otherwise, as shown on the figure's right side. This methodology preserves the network topological structure. Final visualization is obtained by vertex color mapping according to a property or measurements, e.g. node degree.

Here we chose to use molecular dynamics simulation (MDS) [37] to minimize the energy of the system. Although there are many advanced methods to discover the equilibrium states of force-directed systems, MDS is a simple approach and allow us to explore the network interactively, for instance by changing the force constants 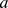 and 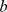
in real time or the even the connection threshold (Fig. 3).

Our algorithm starts by assigning random positions – uniformly distributed over the desired metric space – for each node. Then the MDS equation:
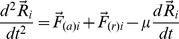
(4)is solved iteratively using the traditional Runge-Kutta [38] method, where the third term acts as a viscosity force with constant 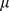 to cool down the system and achieve an equilibrium configuration. The algorithm ends when the nodes start to move slowly because the forces are approaching null (at equilibrium distances), thus attaining a minimal energy state. The parameters 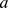 and 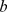 which define the magnitude of the forces also quantifies the scale of the system (see equation 3), while 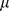 determines the time that the system will take to cool down. In our implementation these parameters can be changed in real time by the user, however we found that 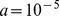, 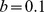 and 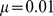 are suitable values for most of real networks. Further optimizations [39] were made rendering this methodology computationally viable for large graph analysis.

### Interactome analysis

Interactome analyses were performed for selected hubs, VIPs and high hubs in the DE and CO transcriptional networks obtained for FS and NFS groups. These analyses were carried out by using an in house free web tool developed by LA Lima & RD Puga – Centro Internacional de Ensino e Pesquisa – Hospital A.C. Camargo and the proteins annotated in MINT, HPRD and IntAct databases (http://bioinfo.lbhc.hcancer.org.br/cgi-bin/interactomegraph/index.cgi). Data analysis and visualization were accomplished through Cytoscape v 2.8.3.

### Histopathology

We used the same previously described histological processing [16], [17], [40] in order to study a larger series of patients: in addition to the 18 cases (6 FS and 12 NFS) previously included in the genomic studies we incorporated other 12 FS and 18 NFS cases, all selected as described above. Briefly, the forty-eight formalin-fixed sclerotic hippocampi were submitted to cryostat sectioning and one out five coronal slices (sixty-micron thick) of the entire hippocampus was stained with cresyl violet (Nissl). The pattern of hippocampal sclerosis was described according to Blümcke et al [41]. Semi-quantitative assessment for granule cell loss (GCL), granule cell dispersion (GCD) and granule cell bilamination (GCB) was made using four hippocampal body slices. Granule cell layer width was measured using ImageJ software (NIH, Bethesda, Maryland, USA). Immunohistochemistry for stargazin followed standard immunoperoxidase protocols for thick floating sections [40].

## Results

**Figure 4:**
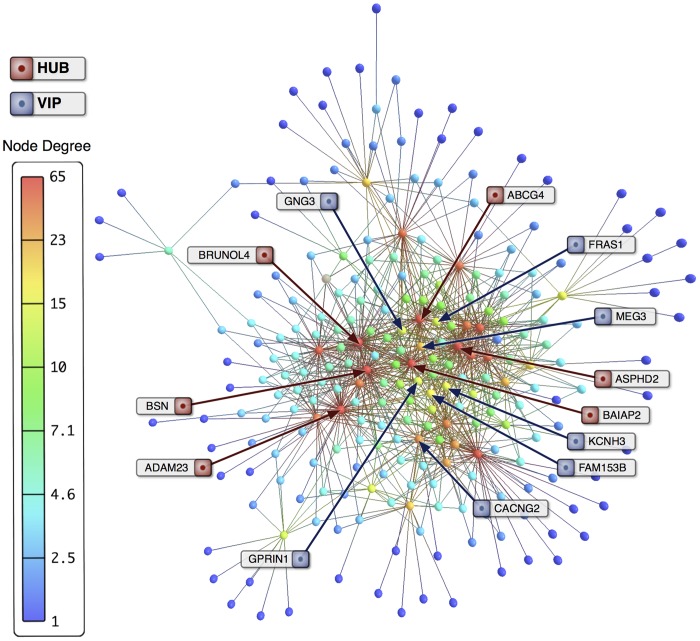
Transcriptional interaction network for FS group. FS transcriptional interaction network based on Pearson's correlation of 307 differentially expressed GO annotated genes (FS-DE). Hubs (red) and VIPs (blue) are identified by their gene symbols.

**Figure 5:**
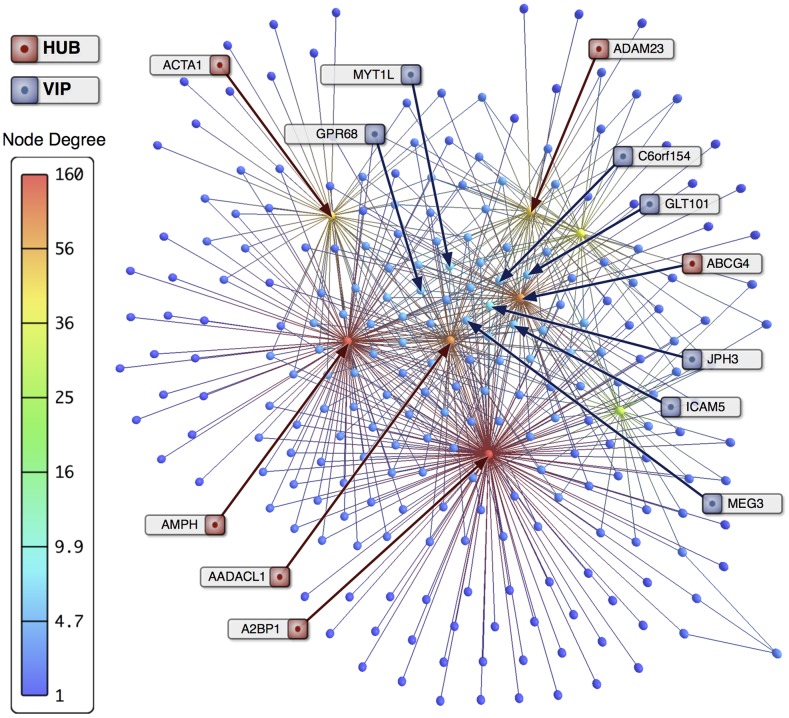
Transcriptional interaction network for NFS group. NFS transcriptional interaction network based on Pearson's correlation of 307 differentially expressed GO annotated genes (NFS-DE). Hubs (red) and VIPs (blue) genes are identified by their gene symbols.

Transcriptional interaction networks for the 307 differentially expressed GO annotated genes (DE networks) were constructed as described in the previous section. Figs. 4 and 5 display the DE networks for FS and NFS groups respectively. Nodes are depicted in gradient color from red to blue according to decreasing node degree values. Complete transcriptional interaction networks (CO) for 15,615 valid GO annotated genes were constructed based on Pearsońs correlation. A link strength cut-off of 0.999 was adopted for FS and NFS networks, yielding a total of 15,585 GO annotated genes for the FS network and 11,233 GO annotated genes for the NFS network. In order to visualize hubs, VIPs and high-hubs in these two CO networks, both were displayed in 3D: videos S1 and S2 show the CO networks for FS and NFS groups respectively. In these videos the nodes are depicted in gradient color (red to blue) according to decreasing node degree values.

**Figure 6:**
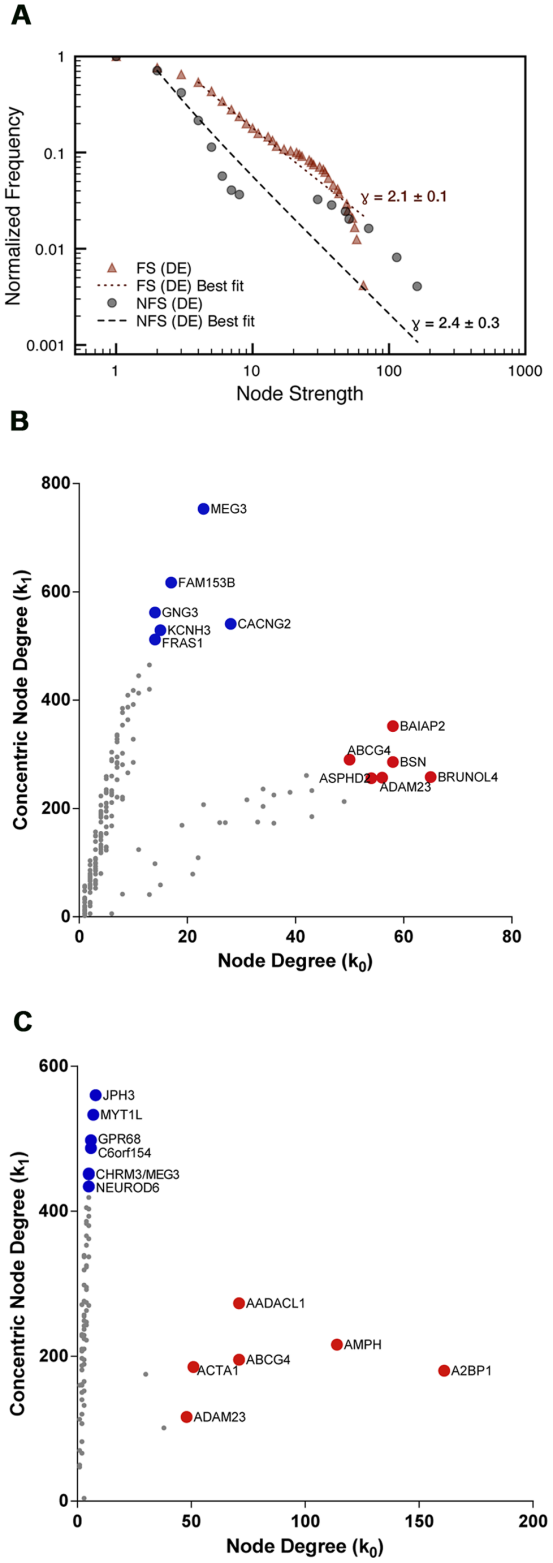
Node distribution and categorization for differentially expressed FS and NFS networks. Normalized degree distribution log-log plot (**A**) for FS and NFS differentially expressed networks, alongside their respective best fitted power law (lines), corresponding to the function 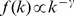. Scatter plot (**B,C**) of node degree (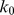) vs concentric node degree (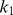) measures of GO annotated genes obtained in the networks for differentially expressed genes (DE). Hubs (red) and VIPs (blue) genes are identified by their gene symbols. 240 nodes for the FS network (**B**) and 246 nodes for the NFS network (**C**).

**Figure 7:**
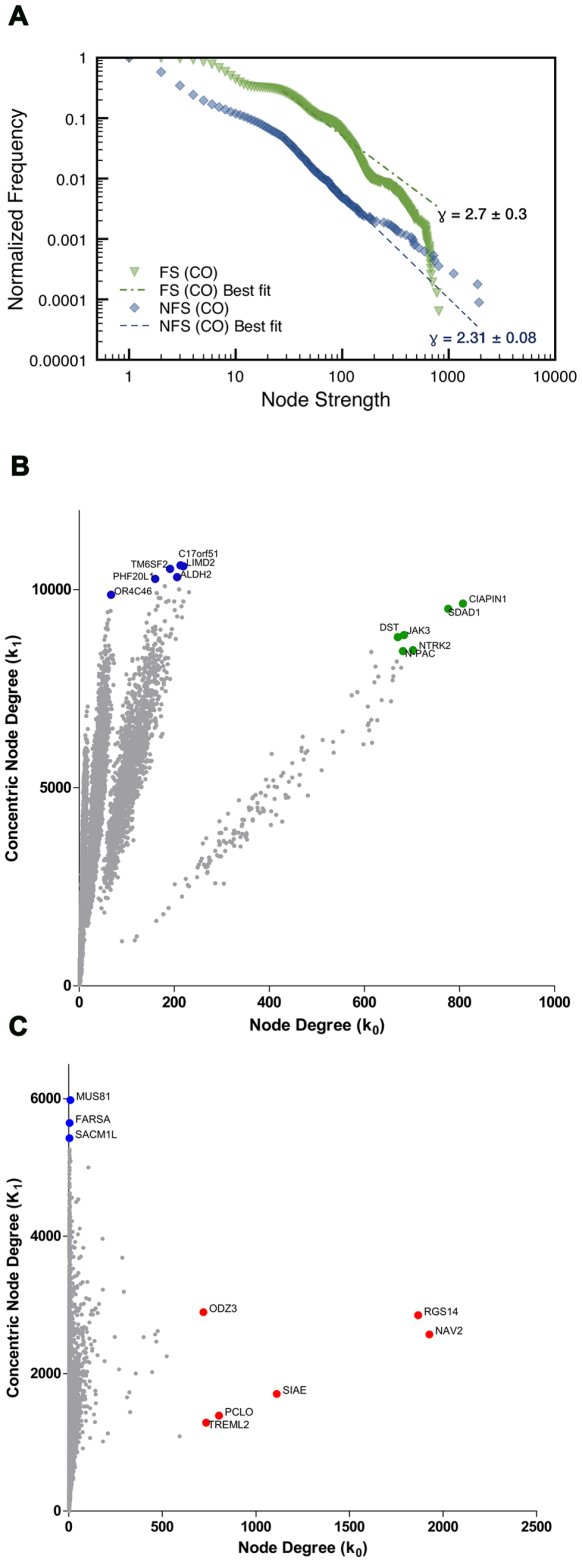
Node distribution and categorization for complete FS and NFS networks. Normalized degree distribution log-log plot (**A**) for FS and NFS complete networks, alongside their respective best fitted power law (lines), corresponding to the function 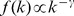. Scatter plot (**B,C**) of node degree (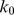) vs concentric node degree (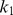) measures of GO annotated genes obtained in the complete transcriptional interaction networks (CO). High-Hubs (green), Hubs (red) and Vips (blue) genes are identified by their gene symbols.15,585 nodes for the FS network (**B**) and 11,233 nodes for the NFS network (**C**).

The frequency distribution of the nodes and their links (Figs. 6A and 7A) and the Kolmogorov-Smirnov test [42] confirms that the networks are indeed scale-free. Concentric categorization (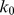 and 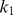) was used for selection of hubs, VIPs and high-hubs, thus permitting the selection of the most connected nodes – hubs, high-hubs – and of the nodes with highest 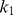 values, i.e. the VIPs. The scatter plots of node degree versus concentric node degree level 1 for nodes of FS (Fig. 6B) and NFS (Fig. 6C) DE networks show the hierarchical categorization of all nodes, hence identifying nodes as hubs (high node degree) or VIPs (high concentric node degree, first level). Table 1 lists the selected genes categorized as hubs or VIPs, their corresponding betweenness centrality values and their biological function. Figs. 7B and 7C show node hierarchical categorization for FS and NFS CO networks identifying nodes as high-hubs (high node degree and high concentric node degree, first level) hubs and VIPs. Table 2 lists the selected genes categorized as high-hubs, hubs or VIPs, their corresponding betweenness centrality values and their biological function.

**Table 1:** Genes categorized as hubs or VIPs in FS and/or NFS transcriptional interaction networks for differentially expressed genes (DE networks).

	FS		NFS		
Gene	Cat^a^	Bt Ct^b^	Cat	Bt Ct	Gene function and/or product [reference]
*BSN*	Hub	0.078		0.001	Bassoon – Speeding of vesicle reloading at excitatory synapses. [44]
*ADAM23*	Hub	0.108	Hub	0.029	Patterning of hippocampal neuronal morphology; interacts with the antiepileptogenic protein LGI1 [50]
*BRUNOL4*	Hub	0.091		0.001	Regulation of genes related to synaptic function [47]
*ASPHD2*	Hub	0.054			Transmembrane protein involved in regulating neuronal motility [53]
*BAIAP2*	Hub	0.075		0.002	Regulation of NMDA receptor-mediated excitatory synaptic transmission, LTP, and learning and memory behaviors [48]
*ABCG4*	Hub	0.055	Hub	0.119	Regulation of neuronal cholesterol efflux [51]
*MEG3*	VIP	0.117	VIP	0.002	Maternally imprinted long noncoding RNA [60] expressed in the pyramidal cell layer of the hippocampus with a putative role in neuronal development/differentiation [117]
*FAM153B*	VIP	0.016		0.000	Family with sequence similarity 153, member B
*GNG3*	VIP	0.014		0.001	G protein gamma 3, abundantly and widely expressed in the brain, required for GABA_B_R regulation of neuronal excitability; modulator of seizure susceptibility [57]
*CACNG2*	VIP	0.102			Codes for stargazin, a receptor regulatory protein that controls the surface and synaptic expression of AMPA type glutamate receptors (AMPARs) [54]. An increase in stargazin expression may be pro-epileptic [16], [55]
*KCNH3*	VIP	0.014			Regulation of excitability in hippocampal pyramidal neurons. Alterations in this gene cause epilepsy [59]
*FRAS1*	VIP	0.042		0.000	Structure and function of basement membranes; cell adhesion [63]
*A2BP1*		0.078	Hub	0.532	Codes for the splicing-regulator Rbfox1 and controls neuronal excitation in the mammalian brain [64]
*AMPH1*		0.069	Hub	0.324	Regulation of endocytic recycling/synaptic vesicle endocytosis [65], [66]
*AADACL1*		0.083	Hub	0.112	Brain serine hydrolase; regulates de novo formation of PAF [67] controlling hippocampal hyperexcitability [68]
*ACTA1*		0.031	Hub	0.102	Actin involved in the regulation of neuronal motility [69]
*JPH3*		0.001	VIP	0.006	Junctophilin 3; involved in the control of hippocampal neuronal excitability [68]
*MYT1L*		0.000	VIP	0.005	Transcription factor involved in neurogenesis, neural cell maturation/functionality; differentiation of dopaminergic neurons [76]
*GPR68*		0.000	VIP	0.004	Proton (pH) sensing G protein-coupled receptor regulating reactive astrogliosis [77]
*C6orf154*		0.043	VIP	0.003	Codifies a leucine rich repeat containing protein (LRR); LRRs are involved in controlling hippocampal synaptic functions [72], [73]
*CHRM3*		0.000	VIP	0.002	Cholinergic neuromodulation of hippocampal circuitry [74]
*NEUROD6*		0.001	VIP	0.003	Neuronal differentiation and stress tolerance [79]

a **Node category: Hub or VIP; ^b^Betweenness centrality measure.**

**Table 2:** Genes categorized as high-hubs, hubs or VIPs in FS and/or NFS complete transcriptional interaction networks (CO).

	FS		NFS		
Gene	Cat^a^	Bt Ct^b^	Cat	Bt Ct	Gene function and/or product [reference]
*CIAPIN1*	Hhub	0.022		0.000	Cytokine-induced inhibitor of apoptosis; protection of hippocampal dopaminergic neurons [90]
*SDAD1*	Hhub	0.02		0.000	Ribosome biogenesis [83] and hippocampal neurite outgrowth [84]
*JAK3*	Hhub	0.017		0.000	Non-receptor tyrosin kinase; involved in proliferation/differentiation of neural precursor cells [91] and survival of CA3 pyramidal neurons [92]
*NTRK2*	Hhub	0.014		0.003	Brain-derived neurotrophic factor (BDNF) receptor; involved in epileptogenesis and in hippocampal synaptic plasticity [85], [86]
*DST*	Hhub	0,012		0,005	Dystonin; cytoskeletal linker protein essential for maintaining neuronal cytoskeleton organization [93]
*N-PAC*	Hhub	0,015		0.000	Regulation of histone demethylation [133]
*C17orf51*	VIP	0,001		0.000	chromosome 17 open reading frame 51
*LIMD2*	VIP	0,001		0,001	Regulation of neural patterning and differentiation of specific axonal projections [95]
*ALDH2*	VIP	0,001		0.000	Protection of hippocampal neurons from oxidative stress [96]
*TM6SF2*	VIP	0,001		0.000	Transmembrane 6 superfamily member 2; Gene ID: 53345
*PHF20L1*	VIP	0,001		0.000	PHD finger; histone reader protein; mediates molecular interactions in gene transcription; involved in neuronal differentiation [58], [99]
*OR4C46*	VIP	0.000		0.000	Putatively involved in regulation of synaptic efficacy [100], [101]
*NAV2*			Hub	0.230	Neurite outgrowth and axonal elongation [102]
*RGS14*		0.000	Hub	0.225	Interfaces hippocampal signalling and synaptic plasticity [103]
*SIAE*		0.000	Hub	0.121	Sialic acid acetyl esterase; sialic acid is involved in the modulation of hippocampal neural and network excitability [104]
*PCLO*		0.000	Hub	0,085	Maintenance of synaptic vesicle clustering; plays a redundant role with BSN [45]
*TREML2*		0,004	Hub	0.076	Ligand in phagocytosis of apoptotic neuronal cells by microglia [106]
*ODZ3*		0,003	Hub	0.070	Regulator of neural connectivity [105]
*MUS81*		0.000	VIP	0.001	DNA recombination repair endonuclease [107] maintenance of ALT cell survival [108]
*FARSA*		0.000	VIP	0.001	tRNA phe; aminoacyl-tRNA synthetases are required for axonal development in hippocampal motor neurons [109]
*SACM1L*		0.000	VIP	0.002	Highly expressed in hippocampus; shares sequence homology with synaptojanins [111] and is involved in astrogliogenesis [113].

a **Node category: High-hub (Hhub), hub or VIP; ^b^Betweenness centrality measure.**

### Transcriptional network analysis of differentially expressed genes (DE networks)

#### FS-DE network

The six selected hubs in the DE network for the FS group (FS-DE network) are genes related to excitatory synaptic function (*BSN, BRUNOL4, BAIAP2*, and *ADAM23*) [43]–[50] or to neuronal homeostasis (*ABCG4* and *ASPHD2*) [51]–[53]. Three out of the six VIPs in FS-DE network – *CACNG2*, *GNG3* and *KCNH3* [54]–[59] – are important neuronal excitability regulators. Another relevant VIP is *MEG3*, a maternally imprinted long noncoding RNA gene involved in neuronal differentiation [60]–[62], which is also a VIP in the NFS-DE network. The other two VIPs are FRAS1, related to basement membranes function and structure, as well as to cell adhesion [63], and FAM153B, that codes for a hitherto uncharacterized protein. The biological functions of these genes are summarized in Table 1.

#### NFS-DE network

In the NFS-DE network five out of the six main hubs (Table 1) are related to synaptic function and hippocampal excitability: ADAM23 [49], [50] and ABCG4 [51], which are also hubs in FS-DE network, *A2BP1* [64], *AMPH1* [65], [66], and AADACL1 [67], [68]. The last hub, *ACTA1*, codes for an actin involved in hippocampal neuronal motility regulation [69]. Three out of the seven NFS-DE network VIPs (Table 1) – *JPH3* [70], [71], *C6orf154* [72], [73] and *CHRM3* [74] – are related to synaptic function and neuronal excitability. The four others VIPs – *MYT1L* [75], [76], *GPR68* [77], *NEUROD6* [78], [79] and MEG3 [60]–[62] – are mostly related to neural cell maturation and hippocampal response to stress.

As a whole, DE networks hubs and VIPs encompass genes related to molecular mechanisms acting mostly on synaptic function and neuronal excitability, but also on neuronal differentiation and motility, and regulation of cholesterol efflux from neurons. All these mechanisms are altered in epilepsy and this topic will be more extensively discussed further in the present paper.

### Complete transcriptional network analysis (CO networks)

#### FS-CO network

The selected genes of the complex network for the FS group (FS-CO) encompass only high-hubs and VIPs (Table 2). Three high-hubs – *SDAD1*, *NTKR2* and *N-PAC* (aliase *GLYR1*) – are respectively involved in brain-derived neurotrophic factor (BDNF)-induced hippocampal neurite outgrowth [80], [81], BDNF modulation of seizure severity and susceptibility [82], [83] and epigenetic regulation of BDNF upon seizures [84], [85]. The other three high-hubs are: *CIAPIM1*, which is linked to apoptosis inhibition [86], [87]; *JAK3* that is involved in the differentiation of neural precursor cells [88] and survival of CA3 pyramidal neurons [89]; and *DST*, acting on neuronal cytoskeleton organization maintenance [90]. Three out of the six VIPs in the FS-CO play relevant roles in neuronal differentiation and neuroprotection (*LIMD2*, *ALDH2* and *PHF20L1*) [91]–[96]. The fourth VIP, *OR4C46*, is a G-protein activated olfactory receptor [97], [98]. The remaining two VIPs in this network, *TM6SF2* and *C17orf51*, code for hitherto uncharacterized proteins.

#### NFS-CO network

The NFS-CO network includes hubs and VIPs, but no high-hubs. All the NFS-CO hubs – *NAV2*, *RGS14*, *SIAE, PCLO, TREML2* and *ODZ3* (Table 2) – are relevant for neuronal functions, particularly in the hippocampus [99]–[103] as it will be further discussed. The three VIPs in the NFS-CO network are associated to compensatory mechanisms, namely: *MUS81*, which is involved in cell survival processes [104], [105]; *FARSA*, a gene related to axonal development of hippocampal neurons [106], [107]; and *SACM1L*, which is highly expressed in the hippocampus and involved in astrogliogenesis [108]–[110].

Taken together, the hubs, high-hubs and VIPs of the CO networks are mostly related to hippocampal neuronal functions, acting either on normal or pathological processes (such as epilepsy) that take place in different hippocampal structures. Complete network analyses bring a potential contribution to understanding neurodevelopmental alterations in epileptogenesis. This issue will be detailed more extensively in the Discussion section.

### Interactome analysis

**Figure 8:**
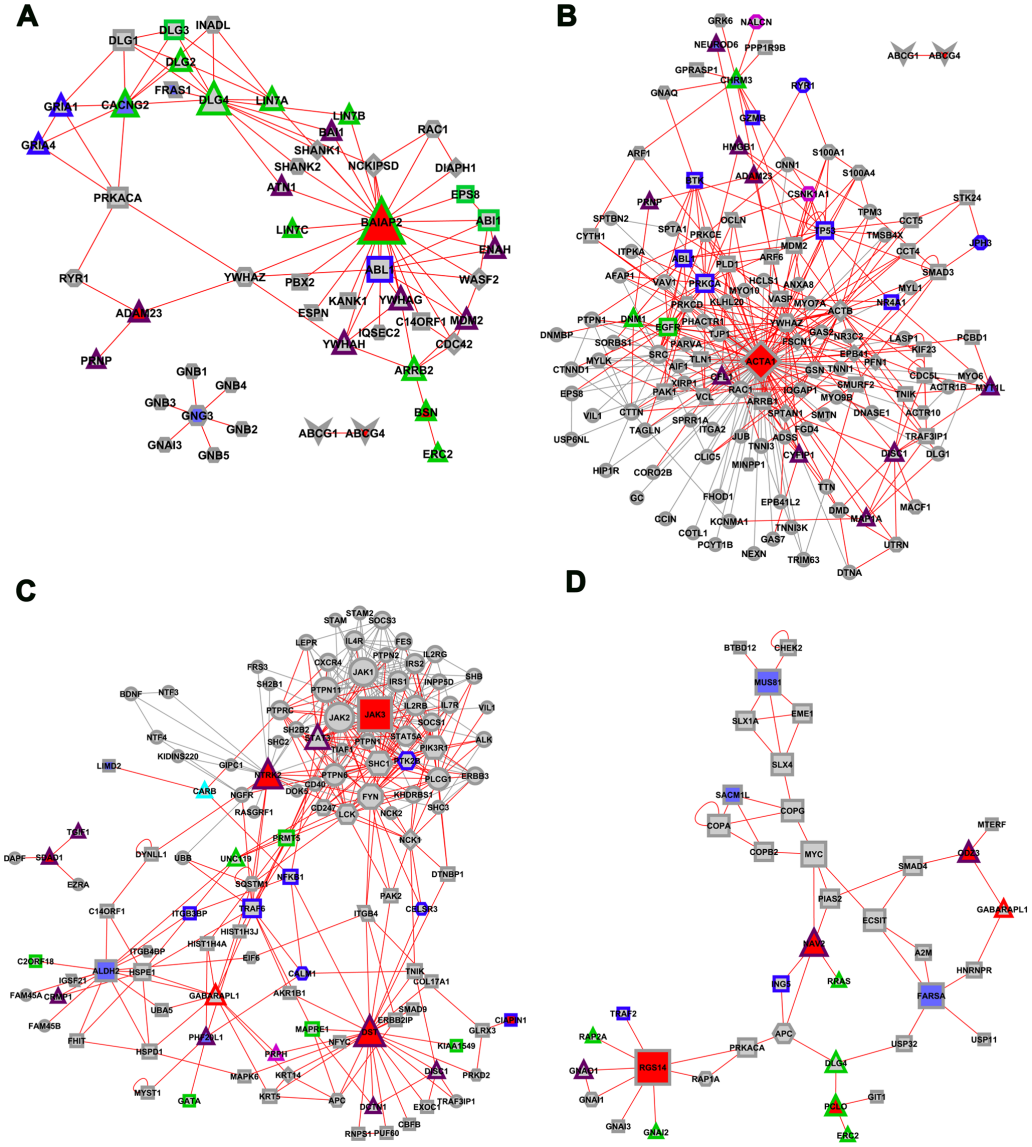
Interactome networks. Interactome networks for DE (**A, B**) and CO (**C, D**) hubs/high-hubs (depicted in red) and VIPs (depicted in blue) using MINT, HPRD and IntAct databases for FS (**A, C**) and NFS (**B, D**) groups. Node shapes and colors represent biological processes, as follows: diamond for actin/cytoskeleton; hexagon for signaling (blue or pink borders stands for neurotransmission or inflammation); octagon for calcium ion binding/transport/homeostasis; octagon with blue or pink border stand for cation channel or cation transport respectively; parallelogram for cell-cell interaction/adhesion; rectangle for cell processes (blue or green borders stand for apoptosis or proliferation); bordered triangles stand for synaptic transmission/SNARE complex (light blue), voltage-gated ion channel activity (pink), GABAergic synaptic transmission (red), synaptic transmission (green) and neuronal differentiation/motility (purple); vee for cholesterol homeostasis; ellipse for other processes. Nodes in red represent high-hubs or hubs in CO or DE transcriptional networks, respectively; Nodes in blue represent VIPs in CO and DE transcriptional networks; Node size is related to node degree (number of links). Links in red represent the first and second hub connections, centered in DE or CO VIPs.

We performed an interactome analysis in order to validate the selected hubs, VIPs and high-hubs in DE and CO networks. Fig. 8 (A–D) shows the interactome networks for DE and CO networks of FS and NFS groups. In NFS-DE interactome network, a hub ACTA1 (also hub in NFS-DE transcriptional network) codifies an actin filament. Conversely, in FS-CO interactome network, a main hub JAK3 (which is a high-hub in FS-CO transcriptional network) codifies a tyrosine kinase. Because these proteins are commonly involved in many cell process pathways, we selected nodes and links for interactome analysis adopting the first and second hierarchical levels centered in the DE or CO transcriptional VIPs (links in red in Figs. 8B and 8C). A description of biological process and/or function based on Gene Ontology database of selected proteins is presented in Tables S1–S4.

### Histopathology

**Figure 9:**
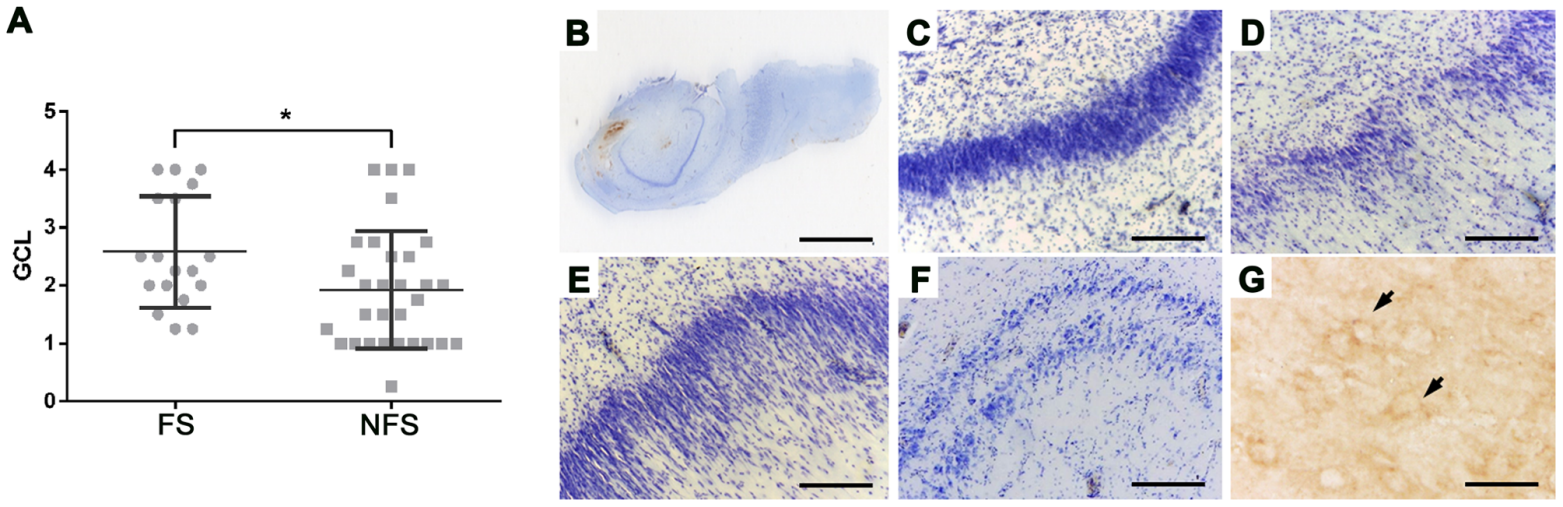
Histopathological results. Histopathological findings in sclerotic hippocampi from patients with epilepsy and semiquantitative analysis of granule cell loss (GCL) in patients with (FS) or without (NFS) febrile seizures as initial precipitant insult. A: semiquantitative analysis of granule cell loss (GCL) in patients with (FS) or without (NFS) febrile seizures as initial precipitant insult (*: p<0.05). B: overview of a histological slice. C: preserved granule cell layer. D: granule cell loss. E: granule cell dispersion. F: granule cell bilamination. G: stargazin-positive cells (arrows). Calibration bars: B 4 mm; C–F 200 um; G 50 um. GCL was graded into the following categories: grade zero (no GCL), grade 1 (mild reduction of neuronal density), grade 2 (moderate reduction of neuronal density), grade 3 (severe reduction of neuronal density), and grade 4 (disruption of neuronal cell layer).

GCL severity was higher (p<0.05) in FS group (Fig. 9A). Differences between FS and NFS groups regarding mesial temporal sclerosis types, GCD severity (Fig. 9E) and GCB occurrence (Fig. 9F) were not statistically significant. Immunohistochemistry confirmed tissue presence of stargazin (coded by the gene *CACNG2*, a VIP in the FS-DE network) and quantitative analysis revealed higher expression of this protein in FS cases when compared to NFS group. Stargazin immunoreactivity was observed in the soma of pyramidal cells of cornu ammonis and subiculum, granule cells and hylar neurons of the dentate gyrus (Fig. 9G). Neuronal processes and perisomatic staining were also observed in principal cell layers. In FS cases immunoreactivity was higher in CA1 (*p* = 0.0004), CA3 (*p* = 0.0007) and granule cell layer (*p* = 0.0012), when compared to NFS group. A detailed analysis of hippocampal neuropathology and immunocytochemistry findings in this series of patients will be published elsewhere.

## Discussion

In this paper we developed a methodology for: i) transcriptional interaction network analysis of differentially expressed non-categorized GO annotated genes; ii) global transcriptional interaction analysis – encompassing all valid GO annotated genes – based on complex network 3D visualization; iii) concentric characterization measuring and node hierarchical categorization. This methodological approach allowed us to: i) construct transcriptional networks for differentially expressed genes without previous gene classification in thematic groups (a bias present in widely used tools, as FunNet [25]); ii) construct global transcriptional networks through new 3D visualization techniques which revealed the global structure of such networks, with emphasis on gene-gene interaction categorization and network topology.

The presented methodology was already been proved to be useful in other knowledge fields [111], however it still novel when analyzing transcriptional networks. The 3D interactive visualization technique allowed us to explore the networks topology and provided good starting points to find interesting features such as the VIP and hub classification.

Here we used this complex network analysis methodology in order to investigate the genomic and molecular mechanisms underlying FS and NFS phenotypes in RTMLE. This approach allowed the analysis of all hippocampal CA3 valid transcripts and the disclosure of a more complete gene-gene interconnectivity pattern in the target tissue. Complex network visualization and analysis, with concomitant node categorization as hubs, high-hubs or VIPs, for each phenotype is critical: in complex diseases, like acquired epilepsy, that are determined by the interplay of genes and environmental factors, the role of the disease-genes (brokers) depends substantially on their gene-gene connectivity and much less on structural gene alterations [15], [21], [22]. Moreover, transcriptional analyses limited to differentially expressed genes do not capture the relevance of interconnectivity based on genes that differ less in their expression and more in their degree of connectivity with other genes. This classification approach presented to be even more informative than the usual way of ranking genes, which relies on their global centrality and immediate number of neighbors, thus revealing only information about the extremes in the scales of the network (respectively, global and strictly local properties).

When a gene-gene interaction network in a disease target tissue suffers a perturbation caused by environmental factors this perturbation triggers a succession of events. In febrile RTMLE, for instance, the release of inflammatory mediators probably drives coordinate changes in the functioning of hundreds of genes in the brain [9]. This process of change is certainly not limited to epileptogenesis but remains throughout the disease duration. In this context, gene functioning reflects both pathogenic and compensatory mechanisms. Correlating changes in transcriptional complex networks with different pathophenotypes may be helpful for finding novel potential therapeutic targets and design intervention strategies.

Joint visualization and analysis of CO and DE networks revealed that DE hubs and VIPs are evenly distributed inside the CO networks (i.e., not as clusters), as shown in videos S1 and S2. Nonetheless, in spite of this shared network topology, the sets of hubs, VIPs and high-hubs of DE and CO networks have somewhat different profiles regarding their biological functions and/or presence in specific molecular pathways, as discussed below.

### DE networks

Considering only the genes with known biological functions in DE networks (i.e. 20 out of 22, see Table 1), four out of the six hubs in FS-DE network are related to synaptic transmission and neuronal excitability, as well as three out of the five VIPs. In NFS-DE network the situation is quite similar: four out of six hubs and three out of five VIPs have this functional profile, totalizing 13 out 20 genes in both networks (*ADAM23* and *ABCG4* are common hubs and MEG3 is a common VIP in DE networks). The remaining hubs and VIPs with known biological function in DE networks (Table 1) are related to: neuronal differentiation (two genes), neuronal motility (two genes), reactive astrogliosis (one gene), neuronal cholesterol balance (one) and cell adhesion (one). All these genes play relevant roles in regulating neuronal functions. Alterations in most of these genes are associated with epilepsy, what makes them potential therapeutic targets. The following discussion is centered in this later issue.

#### FS-DE networks

The first FS-DE hub to considered here, *BSN*, codes for Bassoon, a cytomatrix protein that speeds vesicle reloading at excitatory synapses [44], [43]. *BSN* and the gene *PCLO* (Piccolo), which is a hub in the CO-NFS network, exert a redundant role in maintaining synaptic vesicle clustering [45]. Bassoon interacts with the TrkB/BDNF system in determining cell-type-specific plasticity changes, as seen in mutant epileptic mice lacking Bassoon [112], [113], indicating an interaction between *BSN* and *NTKR2*, a gene that codes for TrkB, the BDNF receptor, and that is also involved in mesial temporal lobe epileptogenesis [82], [84]. *NTKR2* is a high-hub in the CO-FS network, what points out for its relevance in hippocampal CA3 molecular pathways. On the other hand, valproic acid, a commonly used antiepileptic drug, rebalances TrkB and BDNF brain levels in Bassoon mutant mice and reduces epileptic seizures [113]. This underscore *BSN, BDNF and NTRK2* interconnection in epilepsy, corroborated in this study.

Two other hubs, *ADAM23* and *BRUNOL4*, act on neuronal excitability. *ADAM23* controls dendritic arborization in CA1 pyramidal neurons and is a LG1L receptor [50]. *BRUNOL4* (aliase *CELF4*) codes for a RNA-binding protein and is primarily expressed in excitatory neurons – including large pyramidal cells in the cerebral cortex and hippocampus – and regulates excitatory but not inhibitory neurotransmission. This gene regulates translation and local abundance of several synaptic function-associated genes. *BRUNOL4* deficiency causes complex seizure disorders in mutant mice and humans [46], [47]. Both genes play important roles in controlling neuronal excitability. *ADAM23* is an interactor of LGI1, an important antiepileptogenic protein [49], [50] and *BRUNOL4* codes for a RNA-binding protein highly expressed in the hippocampus that regulates several genes related to synaptic functions [47]. The LGI1/ADAM23 (ligand-receptor) complex is considered “an exciting therapeutic target for human epilepsy” [50] and the same can be stated about *BRUNOL4*, since alterations in this gene are involved not only in epilepsy but also in other neurological conditions, such as autism [47].

There are also two relevant nodes common to FS-DE and NFS-DE networks: *ABCG4*, a hub, and *MEG-3*, a VIP, both associated to brain homeostasis and stress responses. *ABCG4* controls neuronal but not astrocytic cholesterol efflux [51]. Changes in brain cholesterol levels during *status epilepticus* occurs in temporal lobe epilepsy [52]. Disruption of brain cholesterol homeostasis occurs in other neurological, neurodegenerative and neurodevelopmental disorders and suggests a potential role of *ABCG4* in brain disorders [51]. *MEG3* (aliase *GTL2*), is a maternally imprinted long noncoding RNA gene [60]. Long noncoding RNAs seem to play relevant functions central nervous system development, homeostasis, stress responses, and plasticity [61], [62]. *MEG3* orthologous gene in mice, *Gtl2*, is strongly expressed in the pyramidal cell layer of the hippocampus and this expression is consistent with a role in neuronal development and differentiation [114].

The remaining FS-DE main hubs are *BAIAP2* and *ASPHD2*. The first is involved in excitatory synaptic transmission and in neurite growth and spine morphogenesis [48] whereas the second codes for an oxidoreductase that regulates neuronal motility driven by transient (mild) oxidative stress [53].

The FS-DE network has three distinctive VIPs, *CACNG2*, *KCNH3* and *GNG3*, with relevant roles in epilepsy. *CACNG2*, which codes for the protein stargazin, is essential for stabilizing diffuse AMPA receptors in the post synaptic density, an important feature of glutamatergic synaptic transmission [54], [115] and genetic absence/alteration enhances thalamic excitability and contributes to epilepsy phenotype [55]. This gene was previously found to be up-regulated in febrile RMTLE [16] and stargazin overexpression in FS patients was confirmed by immunohistochemistry in the present work. Stargazin and AMPA receptor membrane targeting contribute to neuronal hyperexcitability, with a pathophysiological role in the epilepsy phenotype [116]. Stargazin is considered a novel potential target for antiepileptic drugs [117]. *KCNH3*, which codes for a potassium channel Kv7, is a potent regulator of hippocampal and cortical excitability [58], [59] and is also a target of the endogenous antiepileptic neuropeptide somatostatin in hippocampal pyramidal neurons [58], [16]. *GNG3* is widely expressed in the brain and required for GABA_B_R neuronal excitability regulation. The role of GNG3 protein, and of other G-protein subtypes, in neuronal excitability regulation is currently being investigated in animal models: *GNG3* genetic deficiency causes increased susceptibility to seizures in mice [56], [57].

#### NFS-DE network

This network harbors hubs and VIPs with prominent roles in neuronal excitation, synaptic transmission and neuronal differentiation. Two of these hubs, ADAM23 and ABCG4, are also present in the FS-DE network and were commented above. Three out the other four hubs are directly involved in neuronal excitability (the exception is ACTA1, involved in hippocampal neuronal motility regulation [118]). Accordingly, *A2BP1* codes for a RNA-binding protein, Rbfox1, that regulates the alternative splicing of neuronal transcripts involved in synaptic transmission and membrane excitation, some of which involved in epilepsy [64]. The study of post-transcriptional regulation by Rbfox1 is an area of interest for understanding the control of cell homeostasis and misregulation in neurological diseases [64]. This gene probably exerts a role similar to *BRUNOL4* in the FS-DE network, its mutation causing epilepsy [64]. *AMPH1* codes for amphiphysin I, which is an important regulator of synaptic vesicle endocytosis [119] when massive amounts of Ca2^+^ flow into presynaptic terminals, a phenomenon observed in epilepsy [120]. *AADACL1* regulates *de novo* formation of platelet-activation factor (PAF), an inflammation mediator. PAF accumulates in the brain after seizures and causes hippocampal hyperexcitability [121]. *AADACL1* attenuates hippocampal excitability by reducing *de novo* PAF formation [122].

Three of the six NFS-DE VIPs are linked to neural excitability and circuitry control. One of them, *JPH3*, codes for junctophilin 3. Junctophilins 3 and 4 are involved in subsurface cistern formation in hippocampal pyramidal neurons [123] and exert a pivotal role in neural excitability fundamental to plasticity and integrated functions [124]. The two other VIPs are involved in hippocampal synaptic transmission and neuromodulation. *CHRM3*, codes for the muscarinic acetylcholine receptor that confers differential cholinergic modulation to neurochemically distinct hippocampal basket cells subtypes [74]. Early life seizures increase the efficacy of muscarinic receptors coupling to protein G accounting to adult susceptibility to epilepsy, what makes this gene a potential target for novel anticonvulsant drugs [16], [125]. *C6orf154* codes for a leucine rich repeat containing protein (LRR). It was recently discovered that some LRR-containing proteins, as Elfn1, are involved in controlling hippocampal synaptic functions and, moreover, in similar ways as perfomed by LRR containing-Trk neurotrophin receptors and the antiepileptic protein LG1L [72], [73]. Consequently, *C6orf154* is an interesting candidate for further investigations on target-specific synaptic transmission [72].

Among the other three VIPs, two are genes acting on neuronal differentiation and one (*GPR68*) is associated to astroglial cell functions. *MYT1L*, it is an important gene for neurogenesis and neural cell maturation [75], being involved in the maturation of dopaminergic neurons [76] and *NEUROD6* is a gene involved in neuronal stress tolerance in CA1-CA3 pyramidal neurons [78], [79]. *NEUROD6*, a gene responsible for granule cell differentiation in the hippocampus [126], was described as not expressed in heterotopic neurons in epilepsy-induced hippocampal heterotopia [78]. Our patients, in FS and NFS groups, presented heterotopic neurons in the granule cell layer and CA3 expression of *NEUROD6* was found in both groups, being significantly higher in FS cases, as determined by quantitative real-time polymerase chain reaction [16].

### CO networks

There are 19 genes with known biological functions in CO networks (Table 2) and 12 of these genes are involved with hippocampal cell differentiation or neuroprotection. The other seven genes are mostly involved with hippocampal excitability and epileptogenesis. We shall focus here mainly on genes with relevant roles in epilepsy- associated mechanisms.

#### FS-CO network

The selected nodes in this network include just high-hubs and VIPs. High-hubs may play more important roles than hubs and VIPs in transcriptional interaction networks [30]. A very relevant high-hub in FS-CO network is *NTRK2*, which codes for the BDNF receptor TrkB, a therapeutic target candidate. In several MTLE animal models seizures induce a pronounced increase of BDNF expression and enhance the activation of TrkB in the mossy fiber pathway of hippocampus. Transgenic overexpression of BDNF or Trkb increased seizure susceptibility and severity, whereas diminished TrkB signaling reduced epileptogenesis, thus indicating a causal role for BDNF and TrkB in limbic epileptogenesis [82], [87], [127], [128]. BDNF and TrkB are therefore potential targets for antiepileptic and antiepileptogenic therapies [82].

Two among the other five high-hubs, *SDAD1* and *N-PAC* are related to BDNF-driven processes whereas the other three – *CIAPIM1*, *JAK3* and *DST* – are associated to different compensatory mechanisms. Actually, *SDAD1* is involved in ribosomal biogenesis [80], a biological process that has been associated to brain-derived neurotrophic factor (BDNF)-induced hippocampal neurite outgrowth [60]. *N-PAC* (aliase *GLYR1*), regulates histone demethylation [129], a catalytic-activity-based epigenetic drift process that occurs in febrile seizure-induced epileptogenesis [84]. Interestingly, this epigenetic mechanism also regulates the elevation of *BDNF* gene expression after seizures [85]. *CIAPIM1*, codes for anamorsin, a cytokine-induced apoptosis inhibitor [86]. The nuclear translocation of anamorsin is a reactive oxygen species-dependent event and regulates the transcription of several genes involved in the protection of hippocampal dopaminergic neurons [87]. *JAK3* codes for a non-receptor tyrosin kinase and is involved in proliferation and differentiation of neural precursor cells [88] as well as in survival of CA3 pyramidal neurons [89]. *DST*, codes for dystonin, a cytoskeletal linker protein [130], and it is essential for maintaining neuronal cytoskeleton organization.

Four out of the six FS-CO VIPs (Table 2) have known biological functions. Three are involved with hippocampal neuronal patterning and/or differentiation (*LIMD2*, *PHF20L*) and protection of hippocampal neurons against oxidative stress (*ALDH2*), what may be related to compensatory mechanisms, as discussed above. The other VIP, *OR4C46*, codes for an olfactory receptor (OR) and is ectopically expressed in CA3. Although functional interpretation for ectopic OR expression based only on transcription information must be taken with precaution [134], it is relevant that some ORs have been implied in the regulation of synaptic efficiency by modulating Ca^2+^ levels [97], [98]. Moreover, it is worth to consider that: i) several OR genes are expressed in the hippocampus of MTLE patients [132]; ii) genes controlling selective connectivity at dentate gyrus-CA3 and CA3-CA1 synapses map near OR clusters [133], and iii) olfactory sensory neurons are involved with seizure causation in mice [134].

#### NFS-CO network

Some hubs of this network present rather high betweenness centrality values (Table 2), what can be considered a measure of their network essentiality and an indicative of relevant biological roles for these hubs [20], [21]. Three of these hubs probably participate in compensatory mechanisms: *NAV2* is a key player in the processes of neurite outgrowth and axonal elongation [99]; *ODZ3* codes for a teneurin, which is a neuronal connectivity regulator [102]; and TREML2 acts on the phagocytosis of apoptotic neuronal cells by microglia [103]. The other three hubs are linked to synaptic function and neuronal excitability. RGS14 acts at the interface of hippocampal signaling and synaptic plasticity [100]. *SIAE* codes for a sialic acid acetyl estearase. Sialic acid modifications are involved in hippocampal CA3 neural regulation and network excitability [101]. *PCLO* (Piccolo) acts on the maintenance of synaptic vesicle clustering [45]. *PCLO* acts redundantly with *BSN*, which is a hub in FS-DE network, on synaptic vesicle clustering and integrity maintenance [45], [135].

Finally, the three NFS-CO VIPs may be also involved in compensatory mechanisms: *MUS81* codes for a DNA recombination repair endonuclease highly accumulated in nucleoli [104] and is involved in the Alternative Lengthening of Telomeres (ALT)-cell survival process [105], *FARSA* codes for an aminoacyl-tRNA synthetase related to axonal development and *SAMC1L* is highly expressed in hippocampus. This gene regulates Golgi membrane morphology [108], [109], shares sequence homology with synaptojanins [108] and it is involved in astrogliogenesis [110].

Taken together, the results for DE networks reflect a majority of processes linked to synaptic transmission and neuronal excitability, whereas the profile of CO networks show predominance of compensatory mechanisms for the neuronal cell loss/damage associated with epilepsy-induced hippocampal sclerosis [136]. Compensatory mechanisms encompass dentate gyrus neurogenesis [137], synaptic reorganization, axonal sprouting, reactive astrogliosis, and the generation of new excitatory synaptic connections, that can alleviate or aggravate epilepsy [138], [139].

### Interactome validation and histopathology

Interactome analyses (Figs. 8 A–D) validated most of the selected high-hubs, VIPs and hubs obtained through DE and CO transcriptional interaction network analyses: many high-hubs and hubs of transcriptional networks are also hubs in the interactome networks. The DE-FS interactome showed that the majority of the proteins are involved in synaptic transmission and neuronal excitability, whereas the DE-NFS interactome network is more related to neuronal differentiation processes. Both FS-CO and NFS-CO interactome networks showed predominance of pathways linked to neuronal differentiation, synaptic transmission and cell processes associated to compensatory mechanisms, although different subsets of proteins were involved in FS or NFS pathways. Histopathological findings confirmed that stargazin (CACNG2) is significantly more expressed in CA1, CA3 and granule cell layer of FS cases, what is in accordance with our genomic, transcriptional and interactome network data and with our previous work [16]. Finally, it is worth to note that the FS patient's group shows significantly increased granule cell loss (Fig. 9A), probably reflecting enhanced CA3 neuronal excitability and diminished compensatory mechanism activity, as indicated by genomic and interactome data.

### Complex network driven-view of RMTLE

In conclusion, complex network analysis conducted in this study yielded a broad and more detailed view of genomic and molecular mechanisms involved in RMTLE in comparison to analyses centered on differentially expressed genes. Specifically, hubs and VIPs in DE networks are mostly related to neuronal excitability and, in a broad sense, play pro-epileptic roles. Conversely, hubs, VIPs and high-hubs in CO networks are more frequently related to neuronal differentiation, neuroprotection and synaptic function, in a scenario compatible with compensatory mechanisms that may play a reparatory role, but may also be linked to epilepsy pathogenesis [138], [139]. The DE genes with higher connectivity occupy a central position in both DE and CO networks, reflecting their biological essentiality and role in disease [125]. The same network centrality is observed for the hubs, VIPs and high-hubs of CO networks, being consistent with the network disease model [21], where a group of nodes whose perturbation leads to a disease phenotype forms a disease module occupying a central network position [21], [140]. This finding indicates that the probability of exerting therapeutic effects through the modulation of particular genes will be higher if these genes are highly interconnected in transcriptional networks [15].

## Supporting Information

Video S1 Complete transcriptional interaction network for FS based on Pearson's correlation of 15,585 GO annotated genes. High-hubs and VIPs are identified by their gene symbols. FS-DE network hubs and VIPs are also shown.
(DOC)

Video S2 Complete transcriptional interaction network for NFS based on Pearson's correlation of 11,233 GO annotated genes. Hubs and VIPs are identified by their gene symbols. NFS-DE network hubs and VIPs are also shown.
(DOC)

Table S1 Functional description of interactome nodes corresponding to hubs and VIPs in FS transcriptional interaction network for differentially expressed genes (DE).
(DOC)

Table S2 Functional description of interactome nodes corresponding to hubs and VIPs in NFS transcriptional interaction network for differentially expressed genes (DE).
(DOC)

Table S3 Functional description of interactome nodes corresponding to high-hubs and VIPs in FS complete transcriptional interaction network (CO).
(DOC)

Table S4 Functional description of interactome nodes corresponding to hubs and VIPs in NFS complete transcriptional interaction network (CO).
(DOC)
