# Correction: Complex Network Analysis of CA3 Transcriptome Reveals Pathogenic and Compensatory Pathways in Refractory Temporal Lobe Epilepsy

**DOI:** 10.1371/annotation/e9e55996-408c-4660-a0ae-a3597b16f986

**Published:** 2014-01-08

**Authors:** Silvia Yumi Bando, Filipi Nascimento Silva, Luciano da Fontoura Costa, Alexandre V. Silva, Luciana R. Pimentel-Silva, Luiz HM. Castro, Hung-Tzu Wen, Edson Amaro, Carlos Alberto Moreira-Filho

Supplementary Videos S1 and S2 and Supplementary Tables S1 through S4 were incorrectly omitted from the Supporting Information. Please view the Supplementary Videos and Tables at the following links: 

Video S1: http://plosone.org/corrections/pone.0079913.s001.cn.mp4 

Video S2: http://plosone.org/corrections/pone.0079913.s002.cn.mp4 

Table S1: http://plosone.org/corrections/pone.0079913.s003.cn.tif 

Table S2: http://plosone.org/corrections/pone.0079913.s004.cn.tif 

Table S3: http://plosone.org/corrections/pone.0079913.s005.cn.tif 

Table S4: http://plosone.org/corrections/pone.0079913.s006.cn.tif

