# Supplementary material for: Complex Network Analysis of CA3 Transcriptome Reveals Pathogenic and Compensatory Pathways in Refractory Temporal Lobe Epilepsy
Source: PLoS One. 2013 Nov 21;8(11):e79913. doi: 10.1371/journal.pone.0079913 (PMC3836787; doi:10.1371/journal.pone.0079913)
Supplement: Video S2 — Complete transcriptional interaction network for NFS based on Pearson's correlation of 11,233 GO annotated genes. Hubs and VIPs are identified by their gene symbols. NFS-DE network hubs and VIPs are also shown. (DOC) [file pone.0079913.s002.doc]

**Video S2.** Complete transcriptional interaction network for NFS based on Pearson’s correlation of 11,233 GO annotated genes. Hubs and VIPs are identified by their gene symbols. NFS-DE network hubs and VIPs are also shown.
