# Supplementary material for: Complex Network Analysis of CA3 Transcriptome Reveals Pathogenic and Compensatory Pathways in Refractory Temporal Lobe Epilepsy
Source: PLoS One. 2013 Nov 21;8(11):e79913. doi: 10.1371/journal.pone.0079913 (PMC3836787; doi:10.1371/journal.pone.0079913)
Supplement: Table S1 — Functional description of interactome nodes corresponding to hubs and VIPs in FS transcriptional interaction network for differentially expressed genes (DE). (DOC) [file pone.0079913.s003.doc]

**Table S3.** Functional description of interactome nodes corresponding to hubs and VIPs in FS transcriptional interaction network for differentially expressed genes (DE).
